# How Students’ Motivation and Learning Experience Affect Their Service-Learning Outcomes: A Structural Equation Modeling Analysis

**DOI:** 10.3389/fpsyg.2022.825902

**Published:** 2022-04-18

**Authors:** Kenneth W. K. Lo, Grace Ngai, Stephen C. F. Chan, Kam-por Kwan

**Affiliations:** ^1^Service-Learning and Leadership Office, The Hong Kong Polytechnic University, Kowloon, Hong Kong SAR, China; ^2^Department of Computing, The Hong Kong Polytechnic University, Kowloon, Hong Kong SAR, China

**Keywords:** motivation, learning outcome, learning experience, service-learning, SEM

## Abstract

Guided by the expectancy-value theory of motivation in learning, we explored the causal relationship between students’ learning experiences, motivation, and cognitive learning outcome in academic service-learning. Based on a sample of 2,056 college students from a university in Hong Kong, the findings affirm that learning experiences and motivation are key factors determining cognitive learning outcome, affording a better understanding of student learning behavior and the impact in service-learning. This research provides an insight into the impact of motivation and learning experiences on students’ cognitive learning outcome while engaging in academic service-learning. This not only can discover the intermediate factors of the learning process but also provides insights to educators on how to enhance their teaching pedagogy.

## Introduction

The application of motivation theories in learning has been much discussed in the past decades (Credé and Phillips, 2011; Gopalan et al., 2017) and applied in different types of context areas and target populations, such as vocational training students (Expósito-López et al., 2021), middle school students (Hayenga and Corpus, 2010) and pedagogies, including experiential learning and service learning (Li et al., 2016). Motivation is defined in learning as an internal condition to arouse, direct and maintain people’s learning behaviors (Woolfolk, 2019). Based on the self-determination theory, motivation is categorized as intrinsic motivation and extrinsic motivation (Ryan and Deci, 2017). Intrinsically motivated learners are those who can always “reach within themselves” to find a motive and intensity to accomplish even highly challenging tasks without the need for incentives or pressure. In contrast, extrinsically motivated behaviors are motivated by external expectation other than their inherent satisfactions (Ryan and Deci, 2020). To conceptualize student motivation, Eccles et al. (1983) proposed the expectancy-value model of motivation with two components: (a) expectancy, which captures students’ beliefs about their ability to complete the task and their perception that they are responsible for their own performance, and (b) value, which captures students’ beliefs about their interest in and perceived importance of the task. In general, research suggests that students who believe they are capable of completing the task (expectancy) and find the associated activities meaningful or interesting (value) are more likely to persist at a task and have better academic performance (Fincham and Cain, 1986; Paris and Okab, 1986; Kaplan and Maehr, 1999).

Since then, expectancy-value theory has focused on understanding and enhancing student motivation, especially in core academic subjects (Wigfield and Eccles, 2000; Liem and Chua, 2013). Many empirical studies demonstrate that the expectancy-value theory helps understand achievement-related behaviors and performance in key academic subjects in the school curriculum. Studies report that the expectancy and value components are positively related to students’ academic performance. For example Joo et al. (2015) conducted a study on 963 college students enrolled in a computer application course and found that the expectancy component and value component had statistically significant direct effects on academic achievement. Puzziferro (2008) found significant positive correlations between students’ self-efficacy for online technologies and self-regulated learning with the final grade and level of satisfaction in online undergraduate-level courses. Trautwein et al. (2012) conducted a study for 2,508 German high-school students and found that self-efficacy, intrinsic value, utility value and cost can predict academic performance in Mathematics and English. Schnettler et al. (2020) applied expectancy-value theory to study the relationship between motivation and dropout intention. A total of 326 undergraduate students of law and mathematics were studied, and findings showed that low intrinsic and attainment value was substantially related to high dropout intention. These studies argue that the expectancy component, value component and other student experiential variables such as self-regulated learning may positively relate to academic achievement. Recently, this theory has been applied to experiential learning, such as civic education (Liem and Chua, 2013; Li et al., 2016). Results showed that higher expectancy and value beliefs could enhance students’ appreciation and engagement in civic activities, and finally promote the development of targeted civic qualities. This suggests that if expectancy-value theory is applied to service-learning, it would be expected that if students perceive that they are capable of completing the service project (expectancy component) or find the project meaningful (value component), they have higher motivation to engage in the project, and therefore, attain higher learning outcomes.

Students’ motivation in learning can be affected by different factors. These include their emotional, expressive and affective experiences (Pintrich and De Groot, 1990; Deci, 2014), previous learning experiences and culturally rooted socialization, such as gender and ethnic identity (Wigfield and Eccles, 2000). For example, Yair (2000) conducted a study to investigate the effects of instructions on students’ learning experiences. The result showed that structured instructions are better able to improve the learning experiences, which leads to higher motivation of the students. In short, research suggests that students’ motivation affect the academic performance, and motivation itself is impacted by other factors.

Despite all these studies, there has been limited work that applies the expectancy-value theory to study the learning process and understand how the different variables affect students’ motivation and learning outcomes, especially in service-learning. Service-learning is a type of experiential learning that provides a rich set of learning outcomes through applying academic knowledge to engage in community activities that address human and community needs and structured reflection (Jacoby, 1996). Bringle and Hatcher defined academic service-learning as:

*a credit bearing educational experience in which students participate in an organized service activity that meets identified community needs and reflect on the service activity to gain further understanding of course content, a broader appreciation of the discipline, and an enhanced sense of civic responsibility* (Bringle and Hatcher, 1996, p. 5).

This pedagogy helps students translate theory into practice, understand issues facing their communities, and enhance personal development (Eyler and Giles, 1999; Hardy and Schaen, 2000). Previous studies on the benefit of service-learning showed that service-learning could be an effective pedagogy to achieve a wide range of cognitive and affective outcomes, especially on their academic (Giles and Eyler, 1994; Lundy, 2007), social (Weber and Glyptis, 2000), personal (Yates and Youniss, 1996; Billig and Furco, 2002), and civic outcomes (Bringle et al., 2011; Mann et al., 2015). Service-learning is recognized as a high-impact educational practice (Anderson et al., 2019) and it promote positive educational results for students from widely varying backgrounds (Kuh and Schneider, 2008). It is increasingly adopted in universities across the world (Furco et al., 2016; Wang et al., 2020; Sotelino-Losada et al., 2021) and has received significant attention from both academics and researchers in different academic disciplines (Yorio and Ye, 2012; Geller et al., 2016; Rutti et al., 2016), and an increasing number of institutions have formally designated service-learning courses as part of the curriculum (Nejmeh, 2012; Campus Compact, 2016).

Academic service-learning requires students to learn an academic content that is related to a social issue, and then apply their classroom-learned knowledge and skills in a service project that serves the community. In other words, students’ cognitive and intellectual learning is augmented *via* a mechanism that allows them practice of said knowledge and skills (Novak et al., 2007). An example would be learning about energy poverty and solar electricity, and then conduct a service project installing green energy solutions for rural communities in developing countries. Another example is learning about the impact of eye health on academic study, and conducting eye screenings for primary school students. To prepare the students, lectures and training workshops teach students about the academic concepts to equip them with the necessary skills to deal with complex issues in the service setting, and prepare them to reflect on their experience to develop their empathy and build up a strong sense of civic responsibility. The objective is to develop socially responsible and civic-minded professionals and citizens. Therefore, the linkage between academic content, students’ learning and meaningful service activity is critical, as the classroom theory, in a sense, is experienced, practiced and tested in a real-world setting.

Yorio and Ye (2012) suggest that tackling real-life community problems in service-learning leads to increased motivation that can also result in increased cognitive development. However, similar to other educational areas, not much effort has been paid to the “process” by these learning gains are imparted to students. To reveal the mechanism of the learning behavior and provide suggestions for improving the effectiveness of students’ learning, researchers need to investigate the dynamic processes and the influencing factors on how students learn during service-learning. Students do not automatically learn from just engaging in service-learning activities. Instead, how and what students learn depends on different factors. Fitch et al. (2012) suggested using structural equation modeling to develop a predictive model to investigate how students’ initial levels of cognitive processes and intellectual development may interact with the quality of service-learning experiences, and therefore predict cognitive outcomes and self-regulated learning.

Since then, a few studies have been conducted to discover the factors that affect the learning outcomes in service-learning, such as the quality of students’ learning experiences (Ngai et al., 2018), students’ motivation (Li et al., 2016) and students’ disciplinary backgrounds (Lo et al., 2019). Also, Moely and Ilustre (2014) found that the academic learning outcomes were strongly predicted by the perceived value of the service. If students have a clear understanding of the value of the service and acknowledge the benefits to the community, their motivation will increase, which ultimately improves their cognitive learning.

Despite the accumulating evidence suggesting that students’ motivation is an important factor affecting study outcomes, and other research showing that service-learning has positive impacts on students, several research gaps are present. First, there has not been much research using the expectancy-value theory of motivation in service-learning to examine how motivation affects students’ learning from service-learning. Li et al. (2016) explored the effect of subjective task value on student engagement during service-learning and found that the subjective task value of the service played an essential role in their engagement and, therefore, affected their learning. However, this study only focused on the value component of motivation and how this dimension affected students’ engagement, which is correlated to student learning outcomes, but it did not directly study the impact on the learning outcomes. Service-learning, being an experiential learning pedagogy, requires students to actively engage in and reflect on the learning experiences and community needs, then plan and conduct a service project by applying their knowledge (Kolb, 1984). During the project, students interact with the service recipients and instructors to reflect on the assumptions, identifying connections or inconsistencies between their experiences and prior knowledge. This clarification of values and assumptions generate new understandings of the issue, which may lead to changes in the design and execution of the service project. This learning cycle involves a very different set of learning experiences compared to conventional classroom teaching, and thus may impact students differently. This leads us to the second point. As researchers and educators, we must ask how learning occurs and what conditions foster the development. In other words, it is important to examine not only *if*, but also *how*, service-learning affects students’ academic outcomes. Although studies have been conducted to understand the factors influencing students’ learning outcomes, results are far from conclusive.

**FIGURE 1:**
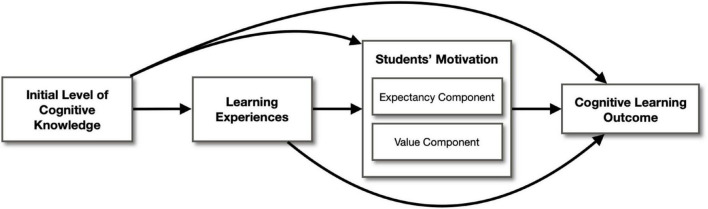
Hypothesized model.

This study aims to fill in these gaps. Grounded on the expectancy-value theory of motivation in learning, the research question would be, “How do students’ learning experiences and motivation affect their cognitive learning outcomes from service-learning?” The hypothesized model is presented in Figure 1, which includes four elements (i) initial level of cognitive knowledge, (ii) the learning experiences, (iii) students’ motivation on the service-learning course, and (iv) the cognitive learning outcome. It posits that students’ cognitive learning outcomes from service-learning are affected by their initial ability, the learning experience, and also mediated by their motivational beliefs about the expectancy component and value component in completing the service-learning tasks. In the service-learning context, if a student perceives that the service project has a high chance of success (expectancy component) and they do find the associated activities meaningful or interesting (value component), then they have higher motivation to engage in the project and thus achieve a higher cognitive learning outcome. In addition, the model hypothesizes that students’ motivation is affected by their learning experiences and their initial level of cognitive knowledge.

To answer the research question, three hypotheses are defined:

1. Based on the preceding literature review, we hypothesize that students’ motivation, both the expectancy and value components, can be impacted by their learning experiences. Also, the initial level of cognitive knowledge of students may have an impact on the motivation (Hypothesis 1).
2. Based on the theoretical framework of the expectancy-value theory of motivation in learning, we expect that students’ motivation, both the expectancy and value components, can positively predict the learning outcomes (Hypothesis 2).
3. Based on the existing literature, we hypothesize that both the students’ learning experiences and their motivation, both the expectancy and value components, directly affect students’ cognitive learning outcome, and motivation can further act as a mediating factor between learning experiences and cognitive outcomes (Hypothesis 3).

## Methodology

The study was conducted at a university in Hong Kong in which service-learning is a mandatory graduation requirement for all full-time undergraduate students. Students have choices over when and which subject to take to meet the requirement. Most of the courses are open-to-all general education type courses, while others are discipline-related subjects restricted to students from particular disciplinary backgrounds or major students. Our study covers 132 of these service-learning subjects offered by 30 academic departments during the 2019/2020 and 2020/2021 academic years. All of the academic service-learning subjects involved in this study carried three credits and followed an overall framework with common learning outcomes standardized by the university, which includes (a) applying classroom-learned knowledge and skills to deal with complex issues in the service setting; (b) reflecting on the role and responsibilities both as a professional and as a responsible citizen; (c) demonstrating empathy for people in need and a strong sense of civic responsibility; and (d) demonstrating an understanding of the linkage between service-learning and the academic content of the subject. All subjects required roughly 130 h of student study effort and were standardized to three main components: (a) 60 h of classroom teaching and project preparation; (b) a supervised and assessed service project comprising of at least 40 h of direct services to the community and which is closely linked to the academic focus of the subject, and (c) 30 h of structured reflective activities. Students’ performance and learning were assessed according to a letter-grade system. The nature of the service projects varied, including language and STEM instruction, public health promotion, vision screening, speech therapy and engineering infrastructure construction. Those projects also covered a diverse range of service beneficiaries, including primary and secondary school children, elderly, households in urban deprived areas, ethnic minorities, and rural communities. Approval for this study was granted by the university’s “Human Subjects Ethics Sub-Committee.”

### Measures

**FIGURE 2:**
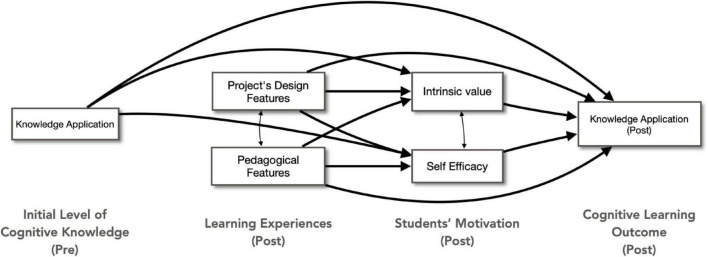
Hypothesized model with measures.

The study employed several quantitative self-report measures to assess students’ learning experiences, learning outcomes, and motivation as described below and shown in Supplementary Appendix 1. Also, hypothesized model with measures was present in Figure 2.

(1) *Students’ learning experiences* was measured by their self-reported experiences regarding the (a) pedagogical features of the course, and (b) design features of the service-learning project. A 13-item instrument was developed in the same university under a rigorous scale development procedure, and students were asked to indicate their experiences after completing the service-learning subject, on a seven-point Likert scale (1 = strongly disagree; 4 = neutral; 7 = strongly agree). All items were written and reviewed by a panel of experts, then a large-scale validation through EFA and CFA was undertaken.

The *Pedagogical Features* dimension included seven items to measure the extent to which students perceived how well they are facilitated and supported in their learning process. This relates to the teachers’ skills in preparing the students for the services, nurturing the team dynamic and assisting the students in reflecting upon the service activities.

The *Project Design Features* dimension included six items to measure to the extent to which students perceived positive experiences during the service project, which is a unique and necessary component of academic service-learning. These features are designed and positioned by the teaching team. Examples include the level of collaboration with the NGO/service recipients and the opportunities for the students to try new things. These experiences are all part of the project design, which, as it is linked to the academic concept covered in the classroom, is controlled by the teacher.

In terms of the construct validity, an exploratory factor analysis (EFA) was conducted with a sample of 11,185 students who completed the service-learning subjects between 2014/2015 and 2018/2019, which yielded a two-factor structure with an 0.81 average factor loading for both aspects without cross-loading at the threshold of 0.30. The reported Cronbach’s α value was 0.90 and 0.89 for pedagogical features and project design features, respectively. Confirmatory factor analysis (CFA) was conducted in this study, and the results showed a good model fit for the two-factor model of learning experiences (χ^2^ = 232.33, *df* = 52, CFI = 0.96, NFI = 0.95, RMSEA = 0.08).

(2) *Students’ motivation* was measured by items taken from the Motivated Strategies for Learning Questionnaire (Pintrich and De Groot, 1990), which included 44 items measuring two main dimensions, (a) Motivational Beliefs (22 items) and (b) Self-Regulated Learning Strategies (22 items). Under motivational beliefs, three sub-dimensions were defined, including intrinsic value, self-efficacy, and text anxiety. Intrinsic value and self-efficacy were corresponding to the value component and expectancy component, respectively, under the expectancy-value model of motivation proposed by Eccles et al. (1983). To align with the institutional service-learning context, an expert review was conducted to select and modify the items. Test anxiety was removed since tests or examinations were not part of the assessment criteria in the service-learning context. One item, “I often choose paper topics I will learn something from even if they require more work,” under the intrinsic value sub-dimension was removed, as the service-learning courses that we are studying require direct services which are connected to tangible community needs and “paper topics” would not be encountered in our context. Wordings from five items were modified to specifically refer to the context for better understanding of students. For example, “class” was changed to “service-learning class” and “class work” was changed to “service project.” The self-regulated learning strategies construct was not included as this study focuses on the causal relationship between learning experiences, students’ motivation, and cognitive learning outcome for engaging in academic service-learning.

After modification, 17 items were selected with eight items from the intrinsic value sub-dimension (value component) to measure the subjective task value of the service-learning subject to the students and nine items from the self-efficacy sub-dimension (expectancy component) to measure the competence belief or expectancy for success in completing the project. Pintrich and De Groot (1990) reported a reliability coefficient of 0.87 and 0.89 for the intrinsic value and self-efficacy, respectively. In this study, a CFA was conducted to ensure the construct validity and a good model fit for the two-factor structure of motivation was found (χ^2^ = 329.05, *df* = 88, CFI = 0.97, NFI = 0.95, RMSEA = 0.08). The average factor loading of intrinsic value was 0.77 and self-efficacy was 0.73.

(3) *Cognitive Learning outcomes* from service-learning was measured by a four-item scale adopted by the Service-Learning Outcomes Measurement Scale instrument (S-LOMS) developed by Snell and Lau (2019). This scale was developed and validated under a cross-institutional research project in Hong Kong. With the localization of the items, the scale contains four dimensions with 11 sub-domains. Students are required to respond to the items on a 10-point Likert-type scale ranging from 1 (strongly disagree) to 10 (strongly agree).

Knowledge application is one of the dimensions that comprise a single cognominal domain to measures the extent to which students are able to understand the knowledge learnt in the service-learning course and apply it to real-life situations. Following the standard approach employed in academic research, the instrument was first developed through review by a panel of experts and focus groups of students. Then, the psychometric properties, including underlying dimensionality and internal consistency, were tested *via* EFA and CFA with a sample of 400 university students from four Hong Kong institutions (Snell and Lau, 2020), reporting a strong internal consistency with a Cronbach’s α value of 0.96. Then, the scale was validated again with another group of students, this time from Singapore (Lau and Snell, 2021). To ensure the construct validity could be maintained, an EFA was conducted for both pre-experience and post-experience data, and the results confirmed a single-factor model with factor loadings over 0.82.

### Participants and Administration

Our survey was administered to all students enrolled in any credit-bearing service-learning subject offered by the institution of study during the 2019/2020 and 2020/2021 academic years. Students were asked to complete a survey both at the beginning and end of the subject. This generally corresponds to the beginning and end of the semester; some subjects ran over multiple semesters. The pre-experience survey was comprised of the cognitive learning outcome (knowledge application) scale while the post-experience survey consisted of items related to their leaning experiences (pedagogical features and project design features), motivation (intrinsic value and self-efficacy) and cognitive learning outcome (knowledge application). Only the pre-experience survey in the fall semester of 2019/2020 was administered *via* paper-based questionnaires. For the rest of the offerings, both pre-experience and post-experience surveys were administered *via* the university online survey platform. To conduct the survey in pen-and-paper format, the course instructors or teaching assistants visited the class to distribute the questionnaires within the first 4 weeks of the semester. For the electronic format, the pre-experience survey was sent to the students by the lecturers within the first 4 weeks of the semester and the post-experience survey was conducted at the end of the subject. For both surveys, email invitations were sent at least twice to follow up with non-respondents to urge them to complete the questionnaire. The collated data was analyzed with the statistical analysis software programs IBM SPSS Statistics (Version 26) and IBM AMOS (Version 26).

### Data Analysis Method

Our data analysis went through the following steps to examine the relationship between students’ learning experiences, motivation and learning outcomes in service-learning, and established the causal effect of the exogenous and endogenous variables.

Means and standard deviations were computed for the data obtained. The reliability of the measures was estimated by the Cronbach’s α values (Cronbach, 1951). Pearson correlation coefficients were calculated to describe the linear association between students’ learning experiences, motivation and learning outcome.

Path analysis in structural equation modeling (SEM) was then employed to examine the effect of initial level of cognitive knowledge, learning experiences and students’ motivation toward learning outcomes using SPSS AMOS 26. SEM is a collection of tools for analyzing connections between various factors and developing a model by empirical data to describe a phenomenon (Afthanorhan and Ahmad, 2014). Path analysis is a special problem in SEM where its model describes causal relations among measured variables in the form of multiple linear regressions. The hypothesized model studied the direct or indirect effects of students’ learning experiences and motivation on their learning outcome. Therefore, the dependent variable was the cognitive learning outcome from service-learning, and the independent variables were their motivation (intrinsic value and self-efficacy) and learning experiences (pedagogical features and projects design features).

The path analysis was conducted through the following steps:

1. Multivariate kurtosis value was computed to confirm the multivariate normality (Kline, 2015);
2. Mahalanobis distances were calculated to determine the outliners (Westfall and Henning, 2013);
3. Goodness of fit of the hypothesized model was tested (Shek and Yu, 2014);
4. R-square (*R*^2^) were computed to illustrate the explained variation; and
5. Standard estimate coefficients (β) of the significant paths were calculated to quantify the “magnitude” of the effect of one variable on another.

## Results

The survey was administered to 8,271 students in the 132 credit-bear service-learning subjects offered during 2019/2020 and 2020/2021. A total of 5,216 and 3,102 responses were received in the pre and post-experience surveys, respectively, making up a response rate of 63.06 and 37.50%. For the paper-based responses, casewise deletion was applied for handling the missing value. For the electronic-based responses, the survey platform would ensures there would not be any missing values. 2,116 (25.58%) valid matched-pair responses were finally obtained and included in the study. A detailed analysis of the respondents’ demographic information reveals that 883 (41.73%) were female and 1,233 (58.28%) were male. Almost half of the students, 988 (46.69%), were from junior years, while 1,128 (53.31%) were from senior years. In terms of the disciplinary background, 608 (28.73%) were from engineering, 530 (25.05%) students from business and hotel management, 475 (22.45%) were studying health sciences, 254 (12.00%) were in hard sciences, and the remaining 249 (11.77%) were in humanities, social sciences, or design. Of the 132 subjects, 46 (34.85%) were from the discipline of health sciences, 31 (23.48%) were from engineering, 27 (20.45%) from humanities and social sciences, 14 (10.61%) from hard sciences, and the remaining 14 subjects (10.61%) were from the business, hotel or design disciplines.

### Descriptive Statistics and Reliability of the Measures

The scale scores were computed by taking the arithmetic mean of the items purported to be measuring the respective constructs. Table 1 presented the minimum, maximum, mean and standard deviation for each measure.

**TABLE 1:** Descriptive statistics and reliabilities.

Dimensions	Measures	α	Min	Max	Mean	Standard deviation
Learning experiences	Project design features	0.88	1.17	7.00	5.49	0.87
	Pedagogical features	0.91	1.43	7.00	5.53	0.88
Students’ motivation	Intrinsic value	0.93	1.25	7.00	5.42	0.85
	Self-efficacy	0.94	1.22	7.00	5.34	0.86
Initial level of cognitive knowledge	Knowledge application (Pre)	0.94	1.00	10.00	6.95	1.31
Cognitive learning outcome	Knowledge application (Post)	0.94	1.00	10.00	7.48	1.38

*N = 2,116.*

Generally, students gave medium to high scores on their learning experiences and motivation. The mean scores on their learning experiences with respect to the project design and pedagogical features were 5.49 and 5.53, respectively. For their motivation measures, the means and standard deviations were 5.42 and 0.85 for intrinsic value and 5.34 and 0.86 for self-efficacy. For the knowledge application learning outcome, students reported mean scores of 6.95 and 7.48, respectively, in the pre- and post-experience survey.

Cronbach’s α estimates were computed for the six measures included in the study to check for internal consistency. The results were also shown in Table 1. The alpha values for the scales on learning outcomes and motivation were over 0.93, which would be classified as having excellent reliability (Kline, 2000). On the other hand, the alpha values of the learning experience measures were 0.88 and 0.91, suggesting good to excellent reliability of these two scales.

### Correlations

The Pearson’s product-moment correlations between the measures were presented in Table 2. All correlations were positive at the 0.01 level, which indicated that the measures change in the same direction: when one increased, the others also tended to increase. In other words, students’ motivation and cognitive learning outcome increased when they had a better learning experience. In general, all scales had a medium to strong association except for the initial cognitive learning scale, which had weak to medium associations with other scales.

**TABLE 2:** Correlation between motivation, learning experiences, and learning outcomes.

Dimensions	Measures	1	2	3	4	5	6
Learning experiences	1. Project design features	–	0.83**	0.74**	0.64**	0.30**	0.64**
	2. Pedagogical features		–	0.76**	0.61**	0.32**	0.65**
Students’ motivation	3. Intrinsic value			–	0.74**	0.35**	0.68**
	4. Self-efficacy				–	0.36**	0.61**
Initial level of cognitive knowledge	5. Knowledge application (Pre)					–	0.41**
Cognitive learning outcome	6. Knowledge application (Post)						–

N = 2,116. **Correlation is significant at the 0.01 level (2-tailed).

Students’ ratings on the project design features were significantly related to the two motivational belief measures, with *r* = 0.74 and 0.64 for intrinsic value and self-efficacy, respectively. Their ratings on the project design features were also significantly related to their initial level of cognitive knowledge (*r* = 0.30) and post-cognitive learning outcome (*r* = 0.64) measures. Similar results were observed for the pedagogical features, where the correlation coefficient with the post-experience cognitive outcome score was 0.65, suggesting a highly correlated relationship. However, the correlation coefficient with the pre-experience score was 0.32, suggesting a rather medium level of association between the two. Significant correlations were found between pedagogical features and intrinsic value (*r* = 0.76) and self-efficacy (*r* = 0.61).

Regarding the correlations between motivation and learning outcomes, a medium association was found between motivation and the initial level of cognitive knowledge with reported correlation coefficients of 0.35 (intrinsic value) and 0.36 (self-efficacy). Significant and high correlations were found between motivation and post-experience cognitive learning outcome, with coefficients of 0.68 (intrinsic value) and 0.61 (self-efficacy).

### Path Analysis in Structural Equation Modeling

A path analysis was conducted to determine the causal effects among learning experiences, students’ motivation and learning outcomes. The models were tested using the maximum likelihood method, which required multivariate normality.

**FIGURE 3:**
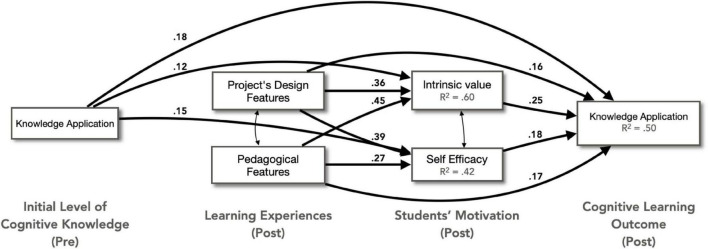
Path diagram between the initial level of cognitive knowledge, learning experiences, students’ motivation, and the cognitive learning outcome.

Multivariate kurtosis value of the observed variables was examined with results ranging from 0.15 to 1.22, suggesting that the variables had a multivariate normal distribution (Kline, 2015). Then, Mahalanobis distances were calculated in AMOS to determine the outliers (Westfall and Henning, 2013), and 60 responses were identified as outliers with a significance level at *p* < 0.001. These responses were therefore excluded from the data. As a result, only 2,056 data points were included in the path analysis. The resulting model was shown in Figure 3, which was consistent with our original conceptual model from Figure 1. The paths shown in the figure were statistically significant at the 0.001 level, and the standardized regression coefficients (β) and explained variation (*R*^2^) were also presented. A chi-square test showed that the estimated model has an acceptable level of goodness of fit [χ^2^ (2, *N* = 2,056) = 225.05, *p* < 0.001]. Table 3 showed the values of goodness-of-fit indices. The CFI, NFI, and GFI values all met the respective criterion for goodness of fit.

**TABLE 3:** Outliers and goodness-of-fit statistics.

Number of outliers	Sample size	CFI	NFI	GFI
60	2,056	0.97	0.97	0.97
Criterion for goodness of fit*		≥0.90	≥0.90	≥0.95

*CFI, comparative fit index; NFI, normed fit index; GFI, Goodness of Fit. Evaluation criteria are determined according to Bentler and Bonett (1980), Bollen and Long (1993), Schumacker and Lomax (2004), and Kline (2015).

The results of the path analysis were consistent with our hypotheses:

*Hypothesis 1: Students’ learning experience and previous cognitive knowledge had a direct effect on motivation.* Intrinsic value was positively predicted by the initial level of cognitive knowledge (β = 0.12, *p* < 0.001), the project design (β = 0.36, *p* < 0.001), and pedagogical (β = 0.45, *p* < 0.001) features of the service-learning subjects as experienced by the students, with a 60% of variation explained. Similarly, self-efficacy was positively affected by the initial level of cognitive knowledge (β = 0.15, *p* < 0.001), the project design (β = 0.39, *p* < 0.001), and pedagogical (β = 0.27, *p* < 0.001) features. These factors explained 42% of the variation of self-efficacy. However, the direct effect of the initial level of cognitive knowledge was much less than the direct effect of the two dimensions of learning experiences.

*Hypothesis 2: Students’ motivation had a positive direct effect on their learning outcome.* Students’ post-experience knowledge application ability is positively predicted by their ratings on intrinsic value (β = 0.25, *p* < 0.001) and self-efficacy (β = 0.18, *p* < 0.001) in completing the service-learning subject.

*Hypothesis 3: Students’ learning experience had a direct effect and an indirect effect mediated by motivation on their learning outcomes.* The total effect (E_*Total*_ = E_*Direct*_ + E_*Indirect*_) of the project design features on cognitive learning outcome was 0.32, with a direct effect (β) of 0.16 and an indirect effect of 0.16 through intrinsic value (E_*Indirect*_ = 0.09) and self-efficacy (E_*Indirect*_ = 0.07). For pedagogical features, the total effect was 0.33 with a direct effect (β) of 0.17 and an indirect effect of 0.16 through intrinsic value (E_*Indirect*_ = 0.11) and self-efficacy (E_*Indirect*_ = 0.05). In total, 50% of the variation in students’ cognitive learning outcomes could be explained by their previous level of knowledge, learning experiences and motivation.

## Discussion

Previous research in academic service-learning in higher education tend to focus on its benefits and impact to students. A number of studies have shown that service-learning is an effective pedagogy for improving cognitive learning outcomes; however, most of these studies were outcome-based rather than process-based (Li et al., 2016). Since the *outcome* of service-learning has been established, we argue that it is now necessary to examine the dynamic *processes* and understand the underlying factors that *produce* these positive learning outcomes. These insights not only provide suggestions for improving the effectiveness of service-learning, but also complete the theoretical framework for understanding the learning behavior in service-learning. Levering on the theoretical support of the expectancy-value theory in motivation, we hypothesized that the expectancy component and value component of students’ motivation play an important role in affecting the cognitive outcome and act as a mediator between the learning experiences and academic outcome.

In line with the research focus, this study aimed to explore the causal relationship between learning experiences, learning motivation, and learning outcomes in the context of academic service-learning. Using a validated, quantitative instrument and analyzing the responses with structural equation modeling showed that in the context of academic service-learning, significant direct and indirect effects were found between initial level of cognitive knowledge, students’ learning experience, motivation and cognitive learning outcomes.

According to the expectancy-value theory introduced by Eccles et al. (1983), motivation is affected by multi-layered factors, including individuals’ perceptions of their own previous experiences, culturally rooted socialization (i.e., gender roles or ethnic identity), and self-schemata (i.e., self-concept of one’s ability or perceptions of task demands). Recent research also found that students’ motivation increases when they gain insight into their values and goals (Brody and Wright, 2004; Duffy and Raque-Bogdan, 2010). This has also been found to be the case in academic service-learning (Darby et al., 2013). Our results demonstrated similar findings in which students’ learning experiences in academic service-learning were a significant determinant of their learning motivation.

From the path analysis, significant direct effects to students’ motivation were identified from students’ initial level of cognitive knowledge and both aspects of learning experiences. These factors positively associated to intrinsic value and self-efficacy, explaining 60 and 42% of the variation, respectively. The effect of the learning experiences were much higher than the effect of the initial level of cognitive knowledge. This indicated that students who had positive learning experiences, regardless of whether the experiences were project- or pedagogically related, were more motivated to learn and were more likely to believe they had the ability to complete the subject. Also, pedagogically related experiences had a slightly larger effect than project design-related experiences on both motivation measures, which implied that preparation and feedback from teachers were more critical with respect to improving students’ motivation than the design of the service project.

Our results suggest that “student motivation” is not static, but could be learned and improved, and the learning experiences played an important role. If educators want better-motivated students, they need to have good interaction with the students, offer necessary support and provide insightful feedback in reflective activities. In the context of academic service-learning, the subject teachers or teaching assistants would achieve best results by working side-by-side with the students throughout the course, including the service project, instead of delegating this component to outside agencies. During the lectures, instructors have to prepare the students appropriately, such as guiding students to understand the linkage between the academic concept and service objective and equipping the students with necessary professional or technical skills. Educators should also regularly conduct reflective activities to cover different aspects of the service-learning course, such as team dynamics, service preparation, community impacts, or personal learning.

On the other hand, even if slightly less critical, the project design features also played an important part. The service project should be designed to be challenging and allow students to have ample direct interaction with the community. Well-prepared students would be more likely to feel competent and confident of success in their project, and challenging but valuable projects that benefit the community and gain the appreciation of the service targets convince students that what they were doing was important and had value. Taking the example of an engineering service project, teachers should allow a certain level of autonomy to the students and challenge them to interact with the collaborating agency or service recipients, understand the needs, and design a tailored solution, rather than asking students to simply replicate a previously designed solution, which may discourage students from engaging in the services, which then leads to a decrease in motivation.

In terms of cognitive outcome, the results of the path analysis indicated that the outcome was affected in three ways, (i) directly through the learning experiences; (ii) directly through the students’ motivation, and (iii) indirectly through the learning experiences with motivation as a mediating factor.

Academic service-learning programs are intentionally designed to have a strong linkage between academic content and service activities. It is known that students do not automatically learn from engaging in service-learning activities. Instead, how and what students learn depends on the quality of their learning experiences (Ngai et al., 2018). Other research has highlighted the importance of the learning experience (Billig, 2007; Taylor and Mark Pancer, 2007; Chan et al., 2019), and showed that they are positively correlated with the learning outcomes (Eyler and Giles, 1999; Joo et al., 2015).

Results of the path analyses showed that the both the pedagogical and project design aspects of the learning experience have similar direct effects and total effects on the cognitive learning outcome, as well as an indirect effect on the outcome through motivation. These findings were consistent with prior studies (Liem and Chua, 2013; Li et al., 2016; Lo et al., 2019) and illustrate the causal relationship between learning experiences, motivations and learning outcomes, which demonstrated the cognitive processes of learning. The standardized beta coefficients further show that the magnitude of the indirect effect was slightly larger than the direct effect, suggesting that the larger impact from the learning experiences is *via* motivation as a mediating factor.

These findings have implications on service-learning practice. One of the differences between academic service-learning and traditional classroom learning is that in service-learning, students need to step outside the classroom and conduct a project to meet identified community needs in real life. Some service projects are delinked from the course material. Sometimes, students are sent out to do piece-meal service or charity work without preparation. Some service projects are over-conceptualized or over-abstracted, for example, having students work primarily on data analysis or reporting. Service-learning teachers should note that both pedagogical and project experiences are equally important. Students needed to understand and relate to the community and individuals they serve, including their needs and their challenges, and to build relationship and empathy with them. Students need also to be equipped with the necessary knowledge and skills for designing and implementing the service, which needs to meet genuine identified needs of the community. Only then do they learn. For example, if students are tackling a challenging project, but they perceive the values and benefits of the services and are well prepared and supported by teachers, and feel connected to and appreciated by the community, they are more likely to recognize the importance of their efforts (value component) and believe that they have the ability to complete the project (expectancy component). This strengthens their engagement and thus they are better able to reflect on their experience and performance. This process positively affects their understanding of the academic content, and therefore, increases their ability to apply knowledge and skills to tackle social issues in real-life service settings.

## Conclusion

We study the causal relationship between learning experiences, students’ motivation, and the cognitive learning outcome in academic service-learning. Decades of research have demonstrated the positive impacts of service-learning on students’ learning, but there has been limited efforts on studying the process and understanding the intermediate factors. Our findings highlight the fact that learning experiences and motivation are key determining factors toward the learning outcome. Motivation in particular is dependent upon the learning experiences, which have not only a direct effect on the outcomes but also indirect influence through motivation as a mediating factor. By applying the expectancy-value theory, this study makes a unique contribution to understanding students’ learning behaviors in academic service-learning. Results show that positive learning experiences can increase the level of expectancy for success and increase the personal value of the project. These can enhance the students’ motivation and engagement in the learning activities, and finally, promote the development of academic learning outcomes.

There are some implications for teachers and practitioners of service-learning. First, students’ motivation can and does change. Second, the learning experience has a strong impact on students’ motivation. Hence, effort should be paid to designing the service project and pedagogical elements. In terms of project design, students need to be intentionally educated, *via* interactions with service recipients and other means of observing or evaluating the impact brought about by their project, the contribution and value of their project to the community. It is also important to expand students’ boundaries with challenging service activities that allow a certain level of autonomy. For example, students conducting public health tests can be tasked with studying the income level and dietary availabilities within the community, and to design some healthy eating menus to share with their community recipients in addition to going through the standardized health test protocol. This challenges students to consolidate and apply their knowledge and allows them some degree of self-directing the design of the projects. In terms of the pedagogical features, teachers and practitioners need to schedule regular – and structured – reflection activities, and make space for good quality interactions with students and ensure that they receive help and support when needed.

It should be stressed that the subjective task-value and expectancy of success are important factors and should be treated with respect. Educators should intentionally design classroom or project activities to highlight these aspects, such as guiding students to reflect on what service-learning and positive citizenship means to them, and how their efforts can contribute to the lives of the underserved in community. These can increase students’ efforts, attention, and persistence in service-learning tasks, which eventually improves their motivation, which can bring positive effects to the learning outcome.

## Limitations and Future Studies

This study has applied expectancy-value theory in understanding the effect of students’ motivation and learning experiences in academic service-learning and shed light on the role of expectancy and value beliefs in the learning outcomes. However, several potential limitations need to be considered when interpreting findings. They also provide directions for future research.

First, the data analyzed in this study were mainly derived from self-report surveys. Although the use of the self-report method may affect the strength of inter-factor relationships examined in this study, we minimize this potential method bias by applying the structural equation analytic technique with a large sample size that purges the measurement of its errors. In future research, additional data sources should be utilized, such as observation from teachers and structured reflective essays, and using different methodological paradigms such as structured interviews or observation. Second, all the data came from one single university in Hong Kong, and the students were enrolled in credit-bearing service-learning subjects within the same curricular framework. The cross-sectional nature of the study is also a limitation. Hence, generalizability of the findings should be viewed with caution. Additionally, after showing that learning experiences are significant predictors to motivation, it would be helpful to understand what *particular* learning experiences have a larger effect on motivation, and whether there are other factors that influence it. Therefore, a future research direction might expand the dimensions of learning experiences to look for causal relationships with students’ motivation. We will also consider other potential variables, such as student demographic data, learning style, personality, or service nature, to enrich our model.

## Data Availability Statement

The raw data supporting the conclusions of this article will be made available by the authors, without undue reservation.

## Ethics Statement

The studies involving human participants were reviewed and approved by the Human Subjects Ethics Sub-Committee, Research and Innovation Office (RIO) The Hong Kong Polytechnic University. The patients/participants provided their written informed consent to participate in this study.

## Author Contributions

KL performed the data collection, statistical analysis, and wrote the first draft of the manuscript. All authors contributed to the conception and design of the study and manuscript revision, read, and approved the submitted version.

## Conflict of Interest

The authors declare that the research was conducted in the absence of any commercial or financial relationships that could be construed as a potential conflict of interest.

## Publisher’s Note

All claims expressed in this article are solely those of the authors and do not necessarily represent those of their affiliated organizations, or those of the publisher, the editors and the reviewers. Any product that may be evaluated in this article, or claim that may be made by its manufacturer, is not guaranteed or endorsed by the publisher.
