# Supplementary material for: How Students’ Motivation and Learning Experience Affect Their Service-Learning Outcomes: A Structural Equation Modeling Analysis
Source: Front Psychol. 2022 Apr 18;13:825902. doi: 10.3389/fpsyg.2022.825902 (PMC9062174; doi:10.3389/fpsyg.2022.825902)
Supplement: Supplementary file 1 [file Table_4.docx]

# Supplementary Appendix I

**TABLE 4 |** Self-reported measures.

| **Measures** | **Dimension** | **Items** |
| --- | --- | --- |
| Students’ learning experiences | Pedagogical Features | My instructors and teaching assistants prepared me appropriately for performing the service. |
|  |  | I could feel the enthusiasm and passion of my instructors and teaching assistants in delivering the subject and the service. |
|  |  | Help and support was usually available from the instructors/teaching assistants/collaborative agency when I needed it. |
|  |  | I benefited a lot from the interaction I had with the instructors, teaching assistants and other students in class. |
|  |  | My team-mates in the SL project were generally motivated and supportive. |
|  |  | I developed a good personal relationship with my teammates. |
|  |  | My teachers/tutors gave me insightful feedback and comments during the reflective activities. |
|  | Project Design Features | I believe that the service I performed in the SL project has benefited the people I served. |
|  |  | I felt that my service was appreciated by the collaborating agency/service recipients. |
|  |  | There were a lot of opportunities for me to meet and interact with the people I served. |
|  |  | The SL project provided challenging and meaningful tasks for me to accomplish. |
|  |  | The SL project challenged me to try things that I had never done before. |
|  |  | In my SL project, I carried out tasks that were mainly designed by me/my team rather than following instructions. |
| Motivated Strategies for Learning Questionnaire (MSLQ) | Intrinsic value | Even when I do poorly on a test I try to learn from my mistakes |
|  |  | I like what I am learning in this class |
|  |  | I prefer class work that is challenging so I can learn new things. |
|  |  | I think I will be able to use what I learn in this class in other classes |
|  |  | I think that what I am learning in this class is useful for me to know |
|  |  | I think that what we are learning in this class is interesting |
|  |  | It is important for me to learn what is being taught in this class |
|  |  | Understanding this subject is important to me |
|  | Self-Efficacy | Compared with other students in this class I expect to do well |
|  |  | Compared with other students in this class I think I know a great deal about the subject |
|  |  | Compared with others in this class, I think I’m a good student |
|  |  | I am sure I can do an excellent job on the problems and tasks assigned for this class |
|  |  | I expect to do very well in this class |
|  |  | I know that I will be able to learn the material for this class |
|  |  | I think I will receive a good grade in this class |
|  |  | I’m certain I can understand the ideas taught in this course |
|  |  | My study skills are excellent compared with others in this class |
| Self-assessment of learning outcomes  (S-LOMS) | Knowledge Application | I know how to apply what I learn in class to solve real-life problems. |
|  |  | I am able to apply/integrate classroom knowledge to deal with complex issues. |
|  |  | I know how to transfer knowledge and skills from one setting to another. |
|  |  | I can make connections between theory and practice. |
